# Paradoxical development of pleural-based masses in patients with pleural tuberculosis during treatment: a clinical observational study in China

**DOI:** 10.1186/s12890-022-01910-6

**Published:** 2022-04-04

**Authors:** Zhengwei Dong, Wei Zhang, Wenwen Sun, Shaojun Zhang, Chenlu Yang, Chunyan Wu, Lin Fan

**Affiliations:** 1 Department of Pathology, Shanghai Pulmonary Hospital, Tongji University School of Medicine, Shanghai, 200433 China; 2 Clinic and Research Center of Tuberculosis, Shanghai Key Lab of Tuberculosis, Shanghai Pulmonary Hospital, Tongji University School of Medicine, No. 507 Zhengmin Road, Yangpu District, Shanghai, 200433 China; 3 Department of Thoracic Surgery, Tongji University School of Medicine, Shanghai Pulmonary Hospital, Shanghai, China

**Keywords:** Tuberculosis, Pleural tuberculosis, Tuberculoma, Risk factors, Pleural effusion

## Abstract

**Background:**

To explored the clinical, pathological, and bacteriological characteristics of pleural-based masses occurred during anti-tuberculosis (TB) treatment in patients with pleural TB.

**Methods:**

Patients referred with newly diagnosed pleural TB were prospectively enrolled into the study. Patients were followed up throughout the treatment, and clinical data were recorded. Percutaneous biopsy and surgical tissues from pleural-based masses were examined histologically and samples sent for PCR. Cytokines in the pleural effusions and clinical factors were collected and compared between different patients.

**Results:**

A total of 122 patients with pleural TB were enrolled, and 34.4% (42/122) displayed newly observed pleural-based mass during the treatment. Twelve cases underwent surgical resection at the 12 ± 0.5 months during the treatment course. Based on the surgical observation, 58.3% (7 /12) were located in pleura, 41.7% (5/12) were located in the lung parenchyma. Pathological observations showed that the pleural-based masses were typed as granulomatous inflammation, fibrous hyperplasia and necrosis. Mycobacterium tuberculosis PCR was positive in 57.1% of the cases (24/42). Any first-line anti-TB drug resistance gene mutations were positive in only 9.5% (4/42). Aside from 12 cases who underwent the surgical operation, 86.7% of the patients (26/30) still had a pleural-based mass at the end of 12 months treatment course. Patients with a pleural-based mass were younger, had a thicker pleural, a higher proportion of pleural adhesive, loculated pleural effusion and residual pleural effusion, and a higher level of LDH, ADA and lower glucose in pleural effusion than those without a pleural-based mass occurrence during the treatment (all Pcorr < 0.05).

**Conclusions:**

Pleural-based masses were observed in about one-third of patients with pleural TB. The masses were in the lung or pleura and were divided into three pathological types.

**Supplementary Information:**

The online version contains supplementary material available at 10.1186/s12890-022-01910-6.

## Introduction

Tuberculosis (TB) remains one of the leading infectious diseases in the world. There were an estimated 10.0 million new cases of TB worldwide in 2020 [1]. Pleural TB is the second most common form of extra-pulmonary TB after extrathoracic lymph node tuberculosis [2, 3]. Pleural-based masses in patient with pleural TB have been observed by imaging [4–8], and the pleural tuberculomas was firstly reported and concerned in 1949 [5]. In clinics, we found a clinical phenomenon in patients with pleural TB: patients with tuberculous effusion had finished the adequate drainage of pleural effusion and initiated the anti-TB treatment when there were no module or mass on chest image. Then masses or nodules closely to pleura on Chest CT or ultrasound were newly observed during the treatment, which were referred as paradoxical development of Pleural-based Masses in Patients with Pleural Tuberculosis during Treatment.

In China, patients with pleural-based mass are frequently identified in clinics. However, published studies only focused on case reports and are mainly reported in Asian regions with a high TB burden [4, 7]. There is no unified treatment regimen for patients when a pleural-based mass is observed by imaging patients receiving anti-TB treatment. This leads to several questions. Why do pleural-based masses occur? How do pleural-based masses present pathologically? Where are pleural-based masses located, in the pleural or pulmonary parenchyma? Should patients with a pleural-based mass receive steroids in addition to standard chemotherapy, or should this be reserved for only those where there is paradoxical enlargement? However, all the above questions remain elusive.

Here, we conducted a clinical observational study to describe the clinical characteristics and explore the pathological findings and features of anti-TB drug resistance gene mutation when pleural-based masses occur to better understand the manifestation of pleural-based masses in the patients with pleural TB.

## Materials and methods

### Study design and patients' information

The study was conducted in a national pulmonary disease specialized hospital, and the patients were mainly from eastern six provinces and one municipality in China. From January 1st, 2017 to June 30th, 2018, patients diagnosed with pleural TB who met the inclusion and exclusion criteria were prospectively enrolled into the study, which was approved by the ethics committee of Shanghai Pulmonary Hospital, Tongji University School of Medicine (No. K16-131). Written informed consent was obtained from all the enrolled participants. Pleural effusion was collected from the included patients and was sent for biochemical and cytokines tests. The patients were followed up throughout the treatment. The treatment outcomes were observed. All the patients with a pleural-based mass were underwent a biopsy via surgical resection or aspiration. The biopsy tissues were tested to assess anti-TB drug resistant genes mutations and pathological observations.

The inclusion criteria were as follows: newly diagnosed as pleural TB and initial treated TB and the pleural effusion could be punctured and obtained; no pleural-based masses or nodules were observed by radiographic appearance (both chest tomography and ultrasound) after effusion drained to dryness (adequate drainage); negative in serum human immunodeficiency virus (HIV), and had no previous history of anti-TB treatment [9].

The excluded criteria were as follows: patients with a malignant tumor; immunosuppressive status; those with other pulmonary diseases; the pleural effusion could not be drawn out or drawn to dryness; patients having characteristics consistent with tuberculous empyema such as having pale creamy or septic pleural fluid [10]; patients had poor compliance to anti-TB treatment; with poor tolerance to chemotherapy resulting in discontinuous treatment; having previously history of TB treatment; having evidence of resistance to first line anti-TB drugs.

The standard of the diagnosis of pleural TB was according to the WHO guideline. Patients were diagnosed with pleural TB at least one of the following criteria were met [11]: (1) identification of Mycobacterium tuberculosis (MTB) in pleural effusion by acid-fast bacilli (AFB) smear; (2) detection of MTB in the pleural effusion by culture or DNA PCR. We considered the result of the RT-PCR as positive when the cycle threshold value was < 37 [12]; (3) typical pathological findings on a pleural biopsy; and (4) positive acid-fast bacteria (AFB) stain or culture in sputum, the chest radiology indicated abnormal lesions compatible with pulmonary TB; and comprehensive clinical standard for the diagnosis of pleural TB (immunology, biochemical or histopathology and responsive to anti-TB treatment, the cutoff value of adenosine deaminase (ADA) in the pleural effusion was 40 IU/L for the diagnosis of pleural TB).

The patients with pleural TB were drained pleural effusion to dryness and were followed up throughout the treatment course. Included patients in this observational study were followed up every month for the examination and treatment. According to the Chinese guideline, the regimen of pleural tuberculosis was 12 months of chemotherapy with 2HREZ/10HRE [13]. All patients were followed up for 12 months after finished the treatment. Outcomes of the patients with pleural-based masses were evaluated and included resolved (lesion resolved by more than 50%), partly resolved (lesion resolved less than 50%) [14, 15], no change and deteriorated (lesion enlarged or diffused).

### Data and sample collection

All the clinical characteristics, including age, symptoms, complications, diagnosis, pleural thickness, pleural nodule, bacteriological tests and treatment regimen were recorded entirely. A pleura thickness > 2 mm, as determined by an ultrasound examination, was considered as pleural thickness [16]. Pleural adhesions were also assessed by ultrasound [17]. The presence of the gliding sign is associated with the absence of adhesions according to Cassanelli et al. [18]. Patients with residual pleural effusion referred to patients drained their effusion by ultrasound as possible as we can, but pleural fluid remained in 34 patients. The patients received a chest CT to observe any changes of the pleural lesions every two or three months during the treatment course after the pleural effusion was drained.

Patients with pleural TB underwent a percutaneous transthoracic puncture to collect the pleural effusion, which was sent to a clinical laboratory for the biochemical testing, and a proportion was frozen at − 20 °C. The biomedical examinations included ADA, lactate dehydrogenase (LDH), protein and glucose. Moreover, IFN- γ, PAI-I, t-PA and TGF-β in the pleural effusion were tested by an enzyme-linked immunosorbent assay (ELISA) (ELISA kit was from eBioscience Corp, San Diego, CA). ELISA was detected on the reader at a wavelength of 450 nm within 30 min after the end of the operation. All the procedures were performed in strict accordance with the product instructions.

### Surgery and biopsy

Surgery was recommended when the pleural-based mass did not resolve after three months of anti-TB treatment and its diameter was larger than 3 cm. Surgeries sometimes were used to exclude malignant tumors. The surgeons decided the operative approach and recorded the precise location of the pleural-based mass during the operation. CT-guided percutaneous biopsies were performed for other patients with pleural-based masses to obtain the tissue. We used disposable core tissue biopsy needles (18G*10 cm) (BRAD Magnum, Mexico, USA) to obtain the biopsies for further testing.

All the samples from surgery and aspiration were sent for pathology and bacteriological tests.

### Pathological observation and immunohistochemistry

The biopsy tissues were prepared for pathologically testing. Formalin-fixed and paraffin-embedding (FFPE) sections were stained with hematoxylin–eosin (HE) and observed by two experienced pathologists. Another pathologist was invited to provide a diagnosis when there was a disagreement between the two pathologists. Ziehl–Neelsen staining was used to examine AFB at 1000X magnification. Masson staining was used to identify collagen deposition. Immunohistochemistry was done using an envision detection system (Leica Biosystems Melbourne Pty Ltd., Melbourne, Australia) and was carried out to estimate the expression of PAI-1(ab226946, 1:200, Abcam, USA) and t-PA (ab157469, 1:50, Abcam, USA) in the pleural-based mass lesions compared to the pleural TB cases without pleural-based masses. As a traditional methodology [19], H-score was used to access the immunohistochemistry results. The score was calculated using the formula 1 × (% of 1 + cells) + 2 × (% of 2 + cells) + 3 × (% of 3 + cells). Zero was for ‘no staining,’ 1 + for ‘light staining visible only at high magnification,’ 2 + for ‘intermediate staining’ and 3 + for ‘dark staining.’

### Detection of anti-TB drug resistance genes mutations in the biopsy tissues

The biopsy tissues were sent for molecular pathological testing, including real-time PCR and PCR-reverse dot blot to detect MTB and drug resistance gene mutations. Paraffin sections 4–5 µm thick (8–10 pieces) were put into an Eppendorf tube. Then, total DNA was extracted using an E.Z.N.A. FFPE DNA Kit (Omega Bio-tek, Inc., USA) according to the manufacturer’s protocol. A total of 4 µl of the extracted DNA was used for real-time PCR to detect the specific gene sequence IS6110 of MTB on a Stratagene Mx3000P QPCR System (Agilent Technologies, Santa Clara, CA, USA). After the amplification, the data was analyzed on Mx3000P. Subject to quality control (no S type amplification curve in the negative control PCR batch; an S type amplification curve in positive control PCR batch with a Ct value < 30; in the standard internal channel, a DNA sample with a Ct value < 45), the MTB was deemed positive if there was an S type amplification curve with a Ct value < 37 in the MTB detection channel. Another 4 µl of the extracted DNA was used for PCR-reverse dot blot to detect rifampicin, isoniazid, streptomycin and ethambutol resistance (rpoB, katG, inhA, embB and rpsL gene accordingly) using a kit for detecting resistance mutations in anti-TB (Yaneng Co, Shenzhen, China) according to the manufacturer’s instructions. A clear blue dot indicated a positive MTB drug resistance gene. Positive and negative controls were included for all the tests described above.

### Statistics

All data record in Microsoft Excel 2010 and analyzed statistically with IBM SPSS v25.0. The continuous data with a normal distribution was compared by Student's t test, if not a Mann–Whitney U test will be applied. Categorical data were compared by chi-square test or Fisher’s exact test. A *P*-value of < 0.05 was considered statistically significant for the comparison of biochemical tests in fluid and clinical factors, multiple comparison on the same cohort take the Bonferroni’s correction. For *p* value corrected for 5 tests of cytokines, *P* < 0.01 were considered for significance. The dot plots were derived from GraphPad prism v7.0.

## Results

### Patients characteristics

**Fig. 1:**
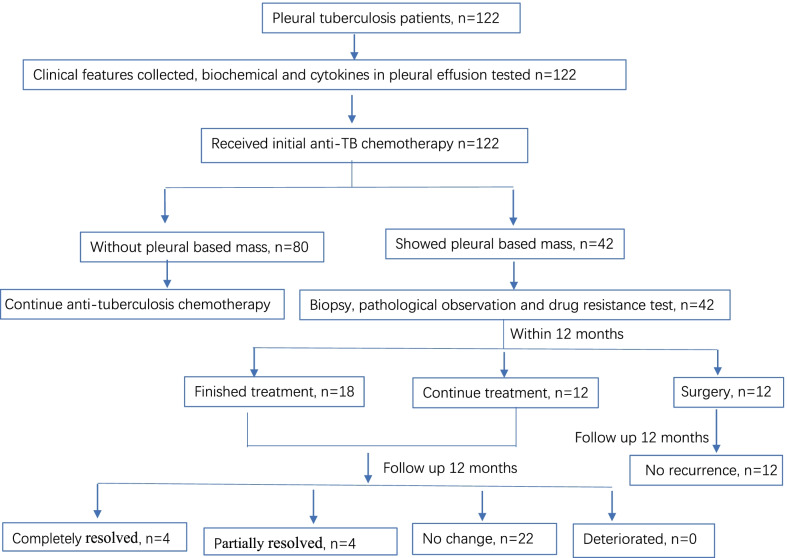
Flow diagram of included participants

A total of 122 patients were finally enrolled in the study, and clinical process were shown in Fig. 1. The median age was 34 (22–59) years old, 66.4% (81/122) were male, 76 patients (76/122, 62.3%) also had pulmonary TB, and 54 cases (54/122, 44.3%) showed pleural thickness, with an average diameter of 3.1 (2.0–4.3) cm. During the treatment, 42 patients developed pleural-based masses, with an occurrence rate of 34.4% (42/122).

All the patients underwent pleural effusion drainage. In 34 cases, the residual pleural effusions were difficult to drain, as by ultrasound at the initial chemotherapy session. The cases with residual pleural effusion had a higher rate of pleural-based mass occurrence than those without residual pleural effusion (p < 0.05). The patients who developed a pleural-based mass were younger and had a larger pleural thickness and pleural adhesion. They also demonstrated a higher proportion of loculated pleural effusion and residual pleural effusion compared with those who did not develop a pleural-based mass. However, the MTB culture positive rates from all specimens were similar for both groups, *P* > 0.05 (see Table 1).

**Table 1:** Comparison of clinical factors between patients with pleural based mass (n = 42) and patients without pleural based mass (n = 80)

Demographic indices	Pleural based mass (n = 42)	No pleural based mass (n = 80)	*P* value
Age, y	29(22–47)	42(27–59)	0.001*
Male	29 (69.0%)	52(65.0%)	0.341
**Clinical factors**			
Plural thickness (millimeter)	33 (78.6%)	21 (26.3%)	0.001*
Pulmonary parenchyma TB involved	28 (66.7%)	48 (60.0%)	0.108
Pleural adhesion	20 (47.6%)	15 (18.8%)	0.001*
Culture positive (pleural effusion)	4 (9.5%)	4 (5.0%)	0.247
Culture positive (sputum)	4 (9.5%)	7 (8.8%)	0.825
Loculated pleural effusion	20 (47.6%)	15 (18.8%)	0.001*
Residual pleural effusion	24 (57.1%)	10 (12.5%)	0.000*

TB = tuberculosis

*P < 0.05

**Fig. 2:**
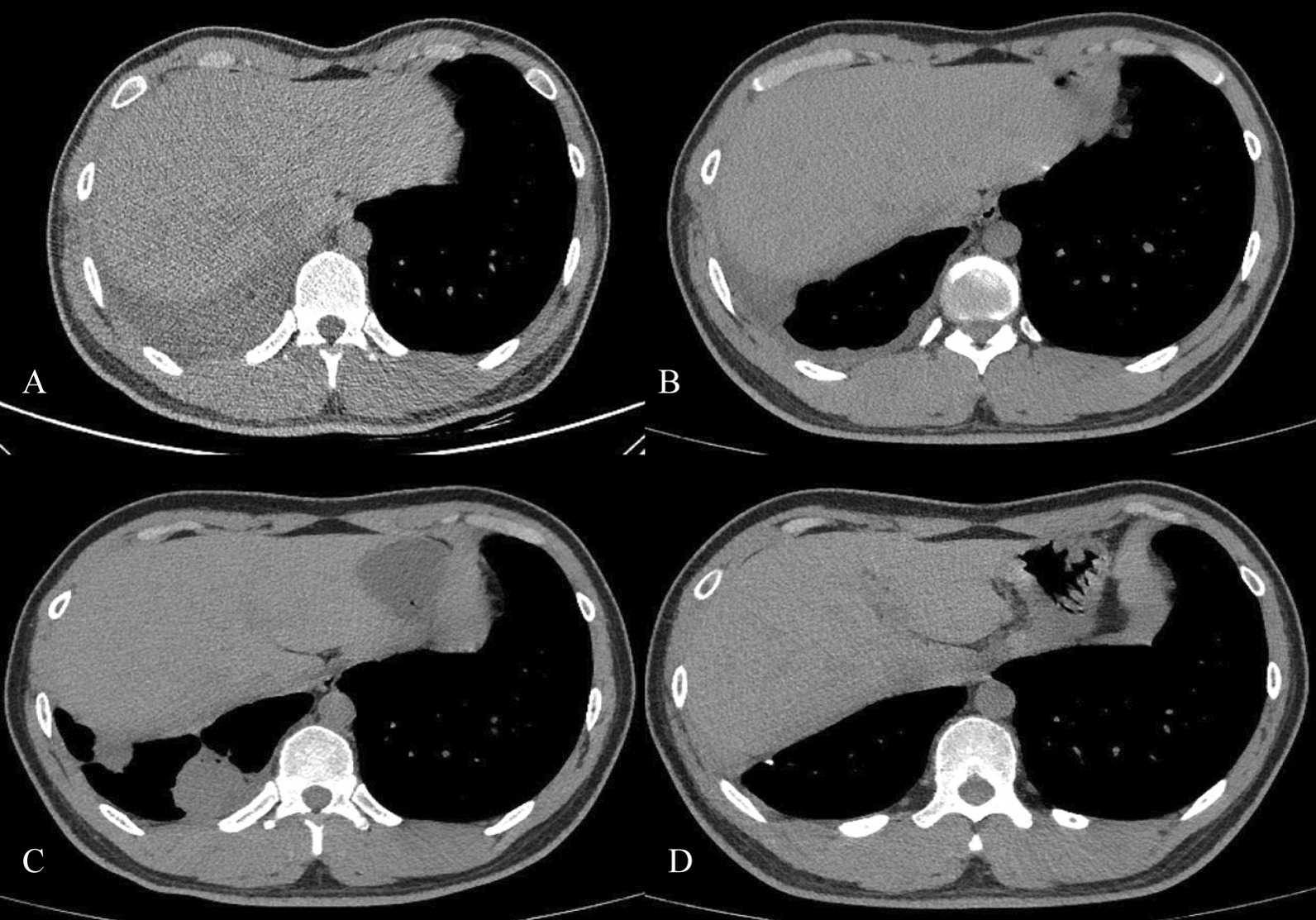
Occurrence and change of pleural-based mass was observed by Chest CT. **A** shows the large amount of pleural effusion in the right chest; **B** shows that pleural effusion was unapparent after draining the pleural fluid and starting the treatment; **C** shows the occurrence of a pleural-based mass in the same site of the right chest after four months of chemotherapy; **D** shows a pleural-based mass resected by surgery, with no recurrence in a year's follow-up

The number of pleural-based masses in pleural tuberculosis patients and the time of pleural-based masses happened were listed in the Table 2. The clinical characteristics and flow diagrams of included cases were shown in Fig. 1. Figure 2 presents the radiological changes during the occurrence of a pleural-based mass.

**Table 2:** Clinical characteristics of pleural-based mass (n = 42)

Pleural based mass	Cases (n, percentage)
Number (N.)	
1	27 (64.3)
2	11 (26.2)
≥ 3	4 (9.5)
Size (in diameter)	
2–10 mm	4 (9.5)
1–3 cm	4 (9.5)
≥3 cm	34 (81.0)
Occurrence time (month)	
< 2	6 (14.3)
2–4	16 (38.1)
4–6	20 (47.6)
Histopathology types	
Granulomatous inflammation	34 (80.1)
Fibrous hyperplasia	3 (7.1)
Necrosis	5 (11.9)

### Biochemical analysis and cytokines expression in the pleural effusion

**Fig. 3:**
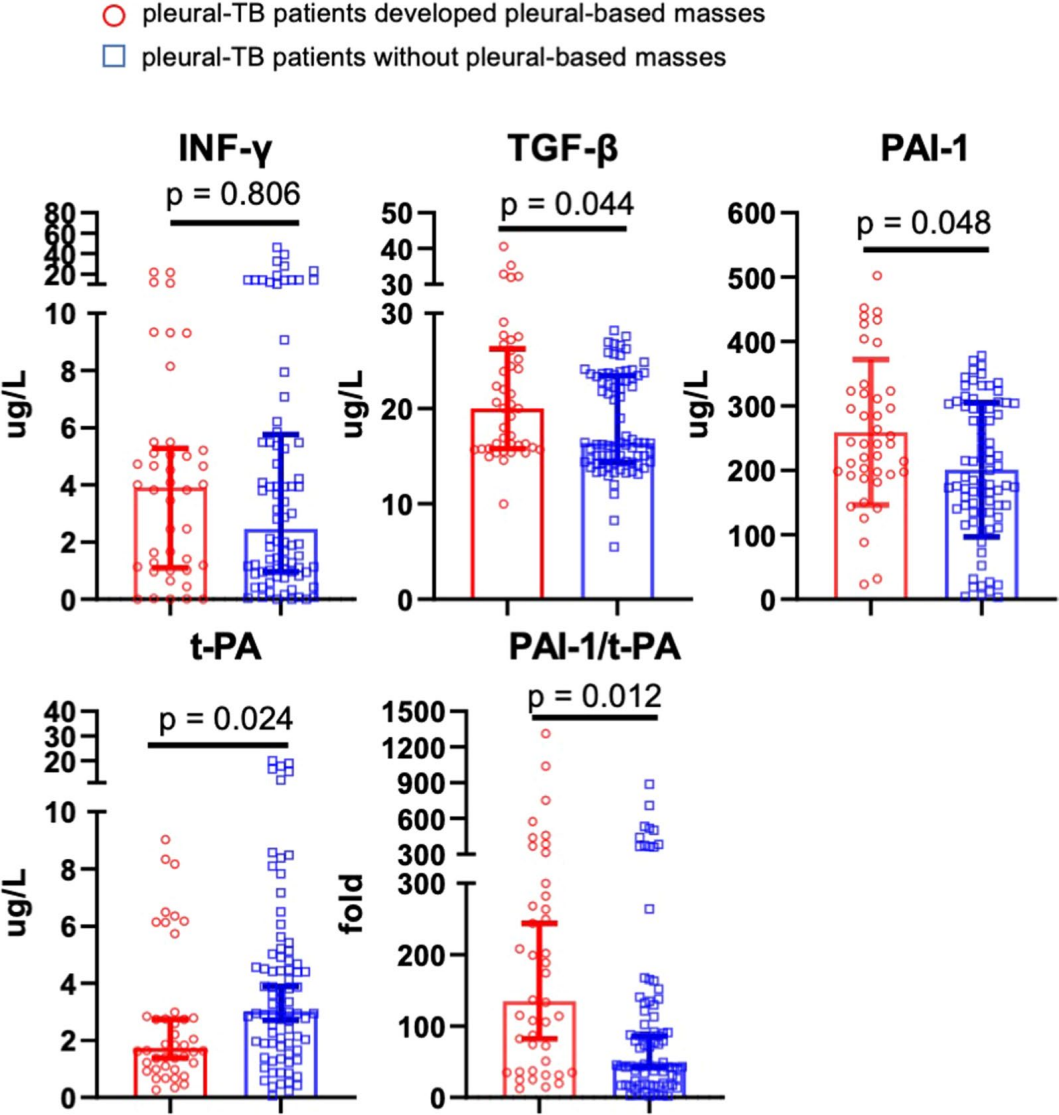
Dot plot (median with Q1–Q3) of five cytokines in pleural effusion between patients with pleural-based mass and patients without pleural-based mass. *Pcorr* value < 0.01 was considered statistical significance

To further explore the characteristics of the patients who developed a pleural-based mass, we tested biochemical markers and cytokines in the pleural fluid at the initiation of the anti-TB treatment. LDH and ADA were significantly higher and glucose was lower in patients with the pleural-based mass patients than those without a pleural-based mass. There was no difference in protein content between the two groups. The data were shown in Table 3 and Additional file 1: Fig. S1. TGF-β, PAI-1 and PAI-1/t-PA were higher and t-PA was lower in the patients with a pleural-based mass than those in patients without a pleural-based mass. However, TGF-β, PAI-1, t-PA and PAI-1/t-PA had no significance between two groups after Bonferroni’s correction in which *P* < 0.01 were considered for significance (Fig. 3).

**Table 3:** Biochemical tests in pleural effusion between patients with pleural based mass and patients without pleural based mass

Biochemical tests in pleural effusion	No pleural based mass median (Q1–Q3)	Pleural based mass median (Q1–Q3)	*P* Value
ADA (IU/L)	69.55 (60.075–83.675)	56.1 (44.625–64.700)	0.001*
LDH (IU/L)	530 (359.75–715.5)	318 (248–419.75)	0.001*
Glucose (mmol/L)	4.7 (3.4–5.8)	4.9 (4.625–6.200)	0.008*
Protein (g/L)	50 (45–54)	47.0 (42–53.75)	0.126

*P < 0.05

### Surgical findings

**Fig. 4:**
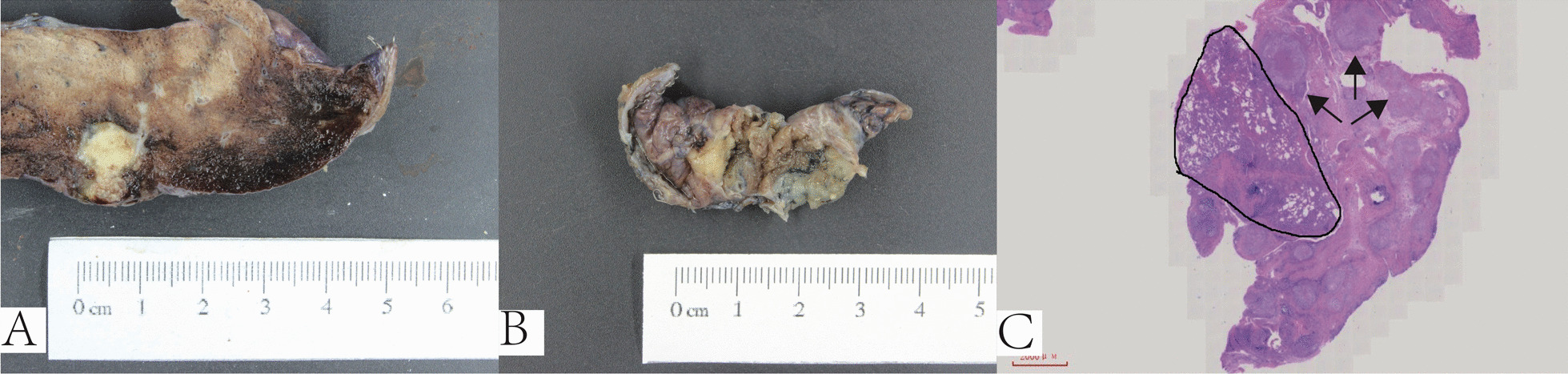
Surgical findings of pleural-based mass. **A** To avoid blood vessels, we take 6 cm lung tissue from this patients. Pleural-based mass was located in lung, and one side of boundary was close to the pleura. **B** It showed pleural-based mass was in the lung parenchyma and pleural, we cannot know where the exact location of lesion in chest during surgery. **C** Pathological morphology of case in the figure B, mass was based on pleura, and there was a clear boundary between pleural based mass and lung parenchyma (black circle). Arrow showed the exact location of granuloma lesion

Among the 42 patients developed a pleural-based mass, 12 (12/42, 28.6%) underwent surgical resection, and 41.7% (5/12) of the pleural-based masses were in pulmonary parenchyma close to the pleura, and 58.3% were located in the pleura based on the surgical findings (see in Fig. 4A–C). Only two pleural-based masses (2/12, 16.7%) showed encapsulated empyema, exhibited as abundant pus wrapped in fibrous tissue. Some (10/12, 83.3%) were tuberculoma-shaped.

### Pathological observations

**Fig. 5:**
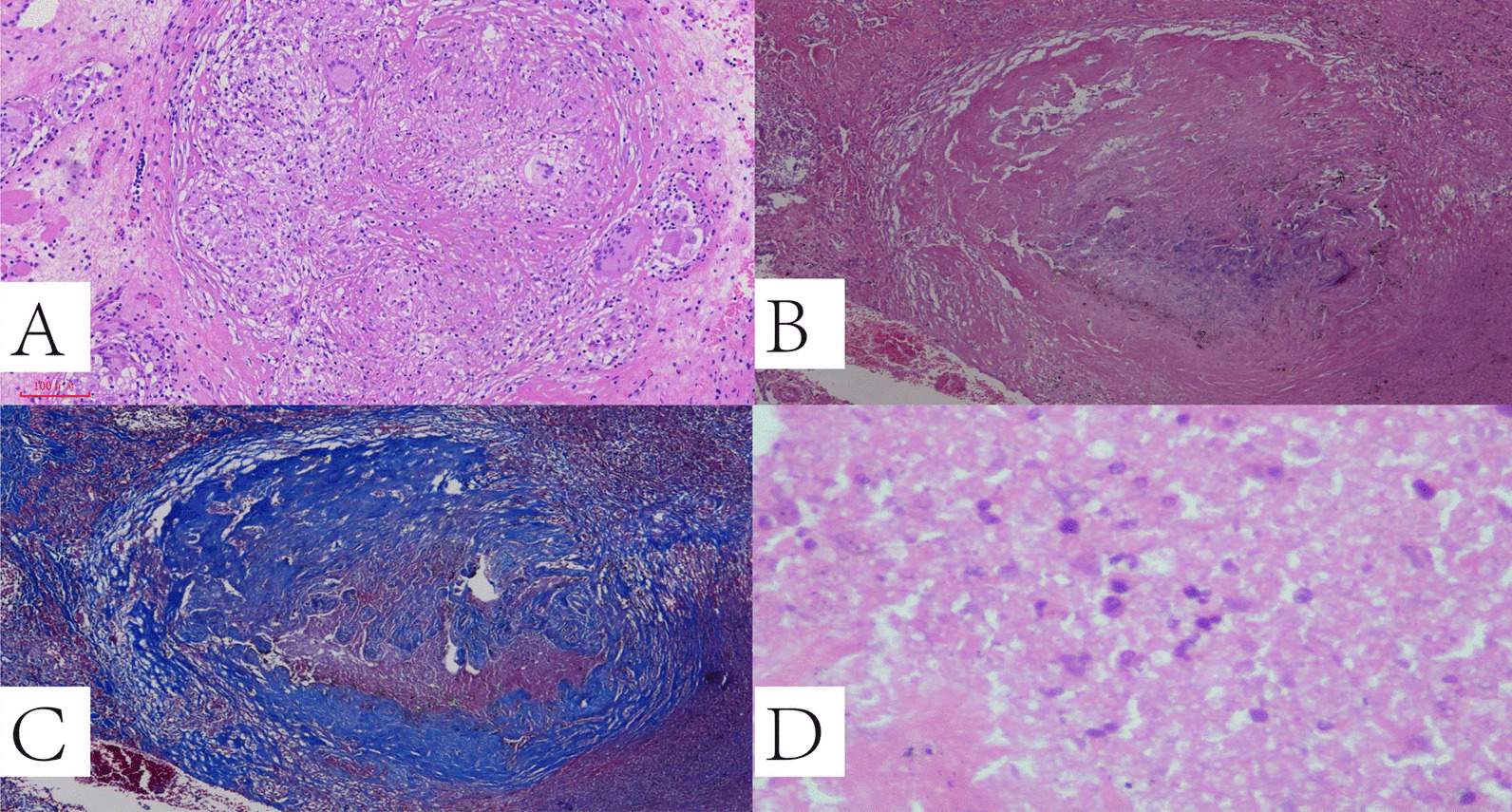
Pathological subtypes of pleural-based mass. **A** Granulomatous inflammation type: composed of inflammatory cells, epithelioid histiocytes, Langhans giant cells and necrosis. HE stain, *100. **B** Fibrous hyperplasia type: lesion mainly consisted of fibroblasts and fibrous proliferation. HE stain, *40. **C** Collagen has been stained to blue, in the same case of B. Masson trichrome stain, *40. **D** Necrosis type: necrosis was dominantly occupied in the lesion. HE stain, *400

Pathological diagnoses were made on 12 surgical and 30 biopsy specimens. According to pathological findings, the pleural-based masses consisted of three pathological types: (1) granulomatous inflammation type; these lesions mainly included inflammatory cells, epithelioid histiocytes, and Langhans giant cells with or without necrosis. Most (34 cases) of the pleural-based mass lesions presented this type; (2) fibrous hyperplasia type; these lesions mainly consisted of fibroblasts and fibrous proliferation without any granulomatous changes. Few cases (3 cases) presented this type; (3) necrosis type; these lesions showed a large area of caseous necrosis with or without an infiltration of inflammatory cells, and fibrous hyperplasia and granuloma were not observed. Necrosis was a dominant morphology, including 2 cases of encapsulated empyema, and 5 cases presented as type 3. (See Fig. 5 and Table 2).

**Fig. 6:**
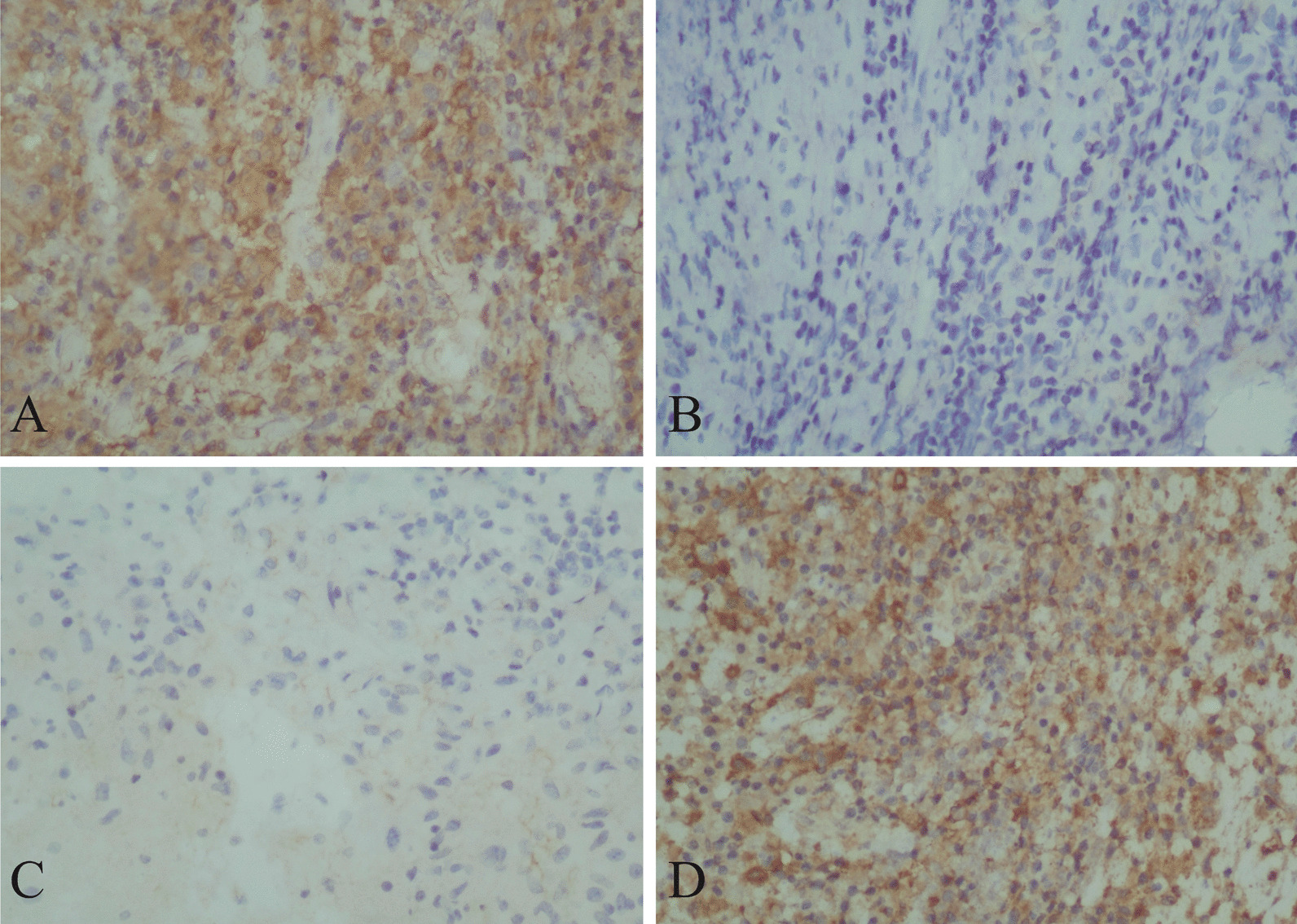
Immunohistochemistry of PAI-1 and t-PA. **A** PAI-1 expression of immunohistochemistry in pleural-based masses. It showed strong stain in cytoplasm of histocytes. **B** PAI-1 expression of immunohistochemistry in patients without pleural-based masses. It was negative in all cells. **C** t-PA expression of immunohistochemistry in pleural-based masses. It only showed very weak stain in cytoplasm of parts of histocytes. **D** t-PA expression of immunohistochemistry in patients without pleural-based masses. Histocytes showed strong stain in cytoplasm

AFB staining was positive in 52.4% of the cases (22/42). Immunohistochemistry expression (Fig. 6) of PAI-1 (50 vs. 20, *p* < 0.05) was higher and t-PA (100 *vs.* 160, *p* < 0.05) was lower in the patients with pleural-based masses compared to the patients without pleural-based masses, which was in accordance with the cytokines change trend in the pleural effusions tested by ELISA.

### Anti-TB drug-resistance genes mutation detections

DNA was extracted from the specimens, and mutation in the rpoB, katG, inhA, embB and rpsL were detected. Among the patients, 57.1% (24 /42) were positive for MTB DNA. Only 9.5% (4/42) detected a mutation in a drug resistance gene, including 1 case with resistance to isoniazid, rifampicin and streptomycin, 2 cases with isoniazid resistance, and 1 case with resistance to rifampicin (see Table 4).

**Table 4:** MTB drug resistance detections in patients with pleural based mass (n = 42)

Type of MTB drug resistance	Cases (n)	Percentage (%)
Isoniazid resistance	2	4.8
Rifampicin resistance	1	2.4
Multidrug resistance of for isoniazid, rifampicin and streptomycin	1	2.4
Total	4	9.5

### Treatment and outcome

We followed all patients with pleural TB, and all the patients without a pleural-based mass were cured after the entire course of chemotherapy. Among the 42 cases with a pleural-based mass, the 12 that underwent surgical resection were cured. In contrast, 73.3% of the cases (22/30) who did not undergo surgical resection showed no change in their pleural-based mass after finishing one year of chemotherapy. Four cases (4/30, 13.3%) were partly resolved, and 4 cases (4/30, 13.3%) were absolved completely. None of patient found their lesions deteriorated. None of the fibrous hyperplasia or necrosis type cases had their pleural-based mass wholly resolved. In the granulomatous inflammation type, 28% (7/25) showed completely or partially resolved lesions. All the patients remained the regimen whether a pleural-based mass occurred or not (see Table 5).

**Table 5:** Status of pleural based mass with different pathologic types after one year's chemotherapy

Status of pleural based mass	Granulomatous inflammation type	Fibrous hyperplasia type	Necrosis type	Total
Completely resolved	4	0	0	4
Partially resolved	3	0	1	4
Stabilization	18	1	3	22
Total	25	1	4	30

Most of the pleural-based masses larger than 3 cm showed no change after the anti-TB treatment course. However, lesions smaller than 1 cm in diameter resolved. In particular, 75% of the small lesions resolved, and 25% were partially resolved after finishing the treatment.

## Discussion

Since 1949, there had been reported that patients with pleural TB were likely to develop pleural-based mass during anti-TB treatment (see Fig. 2) [5, 8]. However, there are limited studies regarding why this happens and the detailed characteristics of the pleural-based mass. We performed an observational study focused on clinical, immunological, bacteriological and pathological findings to further understand the occurrence of pleural-based masses during the treatment.

During the follow-up of patients treated for pleural TB in the present study, 34.4% of patients (42/122) developed pleural-based masses. Twelve cases underwent surgical resection after months of anti-TB treatment due to absorption failure. Among the remaining 30 cases, 73.3% (22/30) had a pleural-based mass that was resolved, even though they finished enough courses of the anti-TB treatment. Secondly, 52.4% (22/42) of the 42 cases were AFB positive within the pleural-based mass, 57.1% (24/42) were MTB DNA positive. Moreover, cases with a sizable pleural-based mass (> 3 cm) had a greater possibility (90.9%, 20/22) of no resolution than those with a smaller mass (≤ 3 cm)(9.1%, 2/22), implying the poor outcome was due to the larger lesions.

We found ADA and LDH was higher in effusion of patients with pleural-based masses, meanwhile glucose was lower. It had been reported that pleural thickness may be related to lower glucose and higher ADA in pleural effusion, and fluid ADA does play an important role in differentiating TB and malignant pleural effusion [20, 21]. The vast majority of tuberculous pleural effusions are based on high protein, a lymphocytosis and low culture positive rate in pleural effusion [22, 23]**.** A low pleural fluid glucose level indicates that the patient probably has infectious disease [23]. We noticed that pleural fluid glucose in patients with pleural-based mass was lower than those without occurrence of pleural-based mass, but seemed higher than patients with mixed other bacterial infection we observed in clinics in which pleural glucose can be obviously low (e.g., as low as 0.03 mmol/L), therefore we speculated that lower fluid glucose in the present study did not exclude the possibility of slightly mixed infection. Although protein content of pleural effusions was thought to be related with pleural thickening, however, we found there was no statistical significance between the two groups in the present study. It might be partly due to small size of study or patient selection bias, the study needs the multicenter, large scale sample clinical trial to verify this conclusion in the future. According to the literature, fibrin deposition and loculation of pleural effusions was related to the imbalance of PAI-1 and t-PA in pleural effusion [24]. Pleural inflammation may increase PAI-1 and decrease t-PA in pleural effusion, it can lead to pleural thickening [25]. Although it seemed like higher TGF-β, PAI-1, and PAI-1/t-PA, lower t-PA were observed in fluid from patients with plural-based masses than those patients without pleural-based masses (*p* < 0.05), these cytokines had no statistical differences after *P* value correction and re-evaluation. We speculated the reason might be limited numbers in the present study, it will be verified in the future study by larger samples.

To further explore the characteristics of the pleural-based masses, we observed their location via surgical resection and pathological findings. Using chest CT or ultrasound, it was difficult to determine the exact location of the ‘pleural mass.’ Lesions adjacent to the pleura as the boundary between the pleural-based mass and lung parenchyma were unclear by imaging. Parts of the lesions needed to be identified under the microscope, as shown in Fig. 4B, C. It suggests that as the pleural effusion clears adhesions might create these masses, and we would carry out a prospective study to find out why pleural-based masses occur in our future work. Through surgical operation and pathological observation, we observed that only 58.3% of 'pleural-based masses' were located in the pleura, and 41.7% cases of the pleural-based mass were located in the lung parenchyma, which suggested that the pleural-based masses were observed by imaging methods and actually were partly located in the pleura and partly located in the lung, indicating pulmonary lesions close to the pleura and involvement with pleural TB.

We further observed the pleural-based masses pathologically by biopsy specimens. There were three pathologically types: granulomatous inflammation, fibrous hyperplasia, and necrosis. The primary pathological change of TB was necrotizing granulomatous inflammation with varying numbers of accompanying non-necrotizing granulomas [26, 27]. Our study showed that granulomatous inflammation was the primary type in the pleural-based masses (81.0%). However, the pathological morphology varied for the different cases. MTB with strong virulence can cause massive necrosis without any granulomas in patients with hypo-immunity, and 11.95 of the cases were as this type (necrosis type) in the present study. In addition to the two above pathological types, 7.1% of the patients exhibited as fibrous hyperplasia, which is more common in a robust immunity environment.

We speculated that the pleural-based masses were likely to be associated with treatment failure because MTB was positive within the lesions even though the patients had taken enough of the chemotherapy course. The treatment failure might be related to low drug concentration levels in plasma [28], decreases in the bioavailability of rifampicin [29], unsteady state of drug exposure [30], Substandard and falsified drugs [31], or the drug resistance occurred during the treatment since patients with evidence of drug-resistance were excluded. And granulomas lesions aroused by macrophage lytic cell death might be involved in this process [32]. The question is now, how to treat the patients when they develop pleural-based masses? Our observation indicated that most pleural-based masses were tuberculoma with positive MTB detected within lesions, and patients with pleural-based masses had more severe inflammation in their pleural cavity. This phenomenon promotes the formation of a granulomatous lesion and the wrapping of fibrous tissue. With MTB, it is difficult to bypass the formation of fibrous tissue and granulomatous lesions, which may just well be the end product of the intense inflammatory reaction and a form of 'cicatricial healing' similar to what is seen with healing in other organs. Therefore, prolonged anti-TB regimens should be required. Whether patients with a pleural-based mass should receive steroids in addition to standard chemotherapy, whether these patients should change the dose of the drugs, and the recommended duration of treatment needs a randomized controlled trial in future.

To answer whether drug resistance plays an essential role in the occurrence of pleural-based masses, we detected the presence of anti-TB drug resistance gene mutations. The results showed that drug resistance was not the main reason for the pleural-based masses because only 9.5% of the cases had drug-resistant gene mutations.

There were some limitations in the present study. It was performed in a referral hospital which was pulmonary specialized hospital, patients admitted into the hospital may have bias, which prefer to be focus on severe and complicated cases, in addition, the study was a singe center study, which might have bias of patients’ selection; and the number we included was limited. The prospective, multi-center study with large samples need to verify the conclusion in the future.

In summary, pleural-based masses were common in patients with pleural TB and had a one-third occurrence rate. Half were located in the lung and presented as granulomatous inflammation, necrosis or fibrous hyperplasia pathologically. Moreover, nearly one-half of cases were AFB positive and had a lower resistance rate within the lesion. The presence of adjoining parenchymal tuberculosis existed in part of paradoxical mass observed in this study was indeed valuable and distinctive.

## Supplementary Information

**Additional file 1: Fig. S1.** Dot plot of biochemical tests with all *Pcorr* < 0.0125).

## Data Availability

The datasets used and/or analyzed during the present study are available from the corresponding author on reasonable request.
