# Supplementary figures and images for: Paradoxical development of pleural-based masses in patients with pleural tuberculosis during treatment: a clinical observational study in China
Source: BMC Pulm Med. 2022 Apr 4;22:126. doi: 10.1186/s12890-022-01910-6 (PMC8981736; doi:10.1186/s12890-022-01910-6)

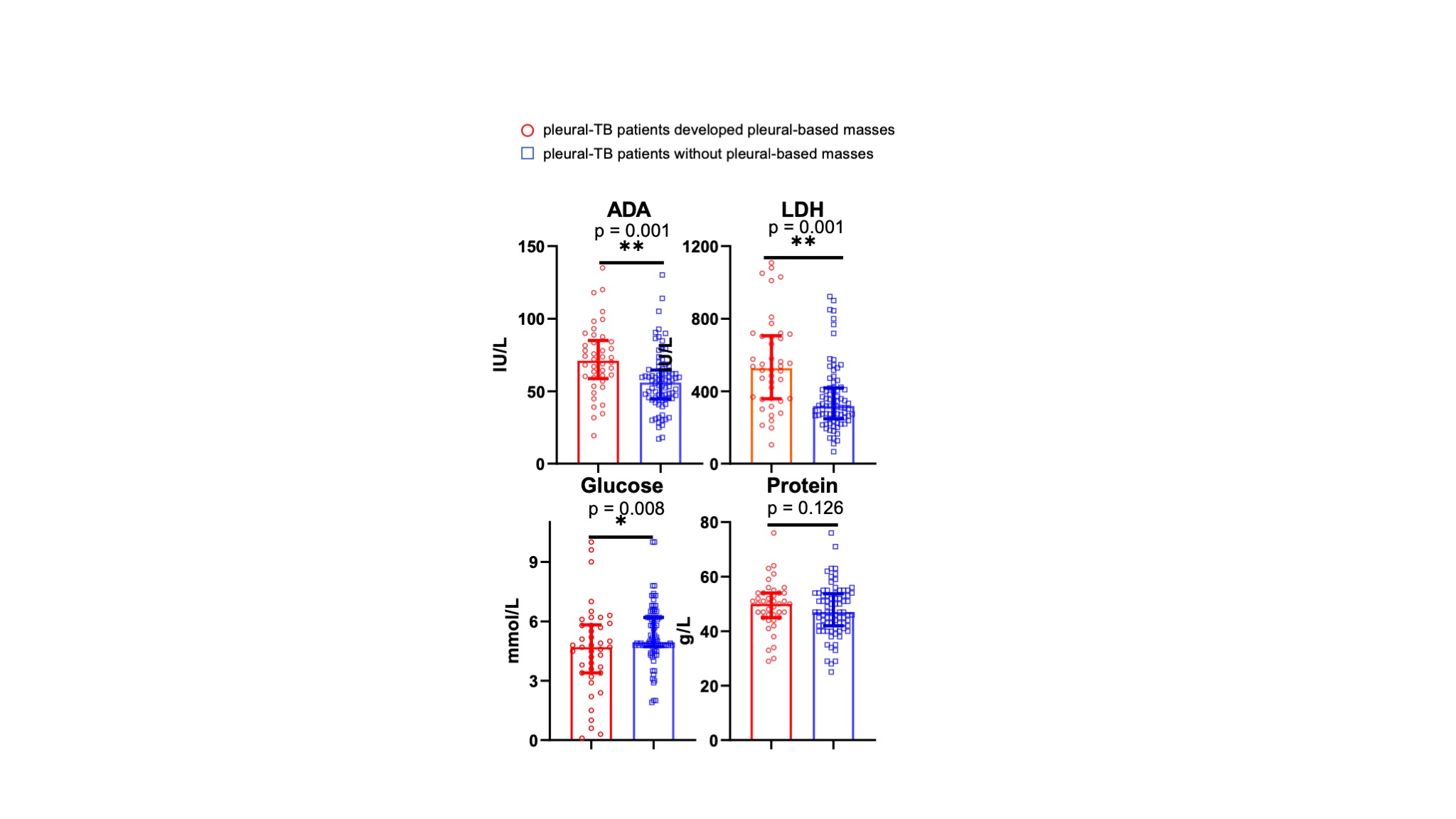

Supplement: Supplementary file 1 — Additional file 1: Fig. S1. Dot plot of biochemical tests with all Pcorr < 0.0125). [file 12890_2022_1910_MOESM1_ESM.jpg]
